# Robotic Complete Mesocolic Excision with Central Vascular Ligation for Right Colon Cancer: Surgical Technique and Short-term Outcomes

**DOI:** 10.1007/s13193-020-01181-9

**Published:** 2020-08-01

**Authors:** C. Ramachandra, Pavan Sugoor, Uday Karjol, Ravi Arjunan, Syed Altaf, Vijay Patil, Harish Kumar, G. Beesanna, M. Abhishek

**Affiliations:** Department of Surgical Oncology, Kidwai Memorial Institute of Oncology, Bengaluru, Karnataka India

**Keywords:** Colonic neoplasm, Right hemicolectomy, Complete mesocolic excision, Central vascular ligation, Right colon cancer

## Abstract

**Background:**

Minimally invasive colorectal surgery has demonstrated to have the same oncological results as open surgery, with better clinical outcomes. Robotic assistance is an evolution of minimally invasive technique.

**Purpose:**

The study aims to present technical details and short-term oncological outcomes of robotic-assisted complete mesocolic excision (CME) with central vascular ligation (CVL) for right colon cancer.

**Methodology:**

Fifty-two consecutive patients affected by right colon cancer were operated between May 2016 and February 2020 with da Vinci Xi platform. Data regarding surgical and short-term oncological outcomes were systematically collected in a colorectal specific database for statistical analysis.

**Results:**

Thirty-seven (71.15%) and 15 (28.85%) patients underwent right and extended right hemicoletomy with an extracorporeal anastomosis. Median age was 55 years. Mean operative time was 182 ± 36 min. Mean blood loss was 110 ± 90 ml. Conversion rate was 3.84% (two cases). 78.84% (41 cases) were pT3 and mean number of harvested lymph nodes was 28 ± 4. 1/52 (1.92%) had a documented anastomotic leak requiring exploratory laparotomy and diversion proximal ileostomy. Surgery-related grade IIIa–IIIb Calvien Dindo morbidity were noted in 9.61% and 1.92%, respectively.

**Conclusion:**

Robotic assistance allows performance of oncological adequate dissection of the right colon with radical lymphadenectomy as in open surgery, confirming the safety and oncological adequacy of this technique, with acceptable results and short-term outcomes.

## Introduction

Colorectal cancer (CRC) ranks third in terms of incidence but second in terms of mortality. Globally, 1.8 million new CRC cases and 881,000 deaths are estimated to occur in 2018, accounting for about 1 in 10 cancer cases and deaths [1]. In the USA, in 2020, it is projected that 147,950 individuals will be newly diagnosed with CRC, including 104,610 cases of colon cancer and 43,340 cases of rectal cancer [2].

Minimally invasive surgery has revolutionized the practice of CRC surgery. There is a plethora of evidence to support the potential benefits of laparoscopic surgery, which includes shorter length of stay, less pain, lower conversion rates and equivalent oncologic outcomes in CRC [3–12]. These trials, performed before complete mesocolic excision (CME) in conjugation with central vascular ligation (CVL), had emerged as a standard procedure.

The key steps of CME+CVL technique emphasizes en-bloc resection of the tumour and its surrounding soft tissue by sharp dissection of the visceral plane from the parietal fascia layer, along with the entire regional mesocolon as a single and intact unit, with dissection of the vessels at their origin to maximize the vertical lymph node yield and an appropriate length of the colon to remove longitudinal pericolic lymph nodes to maximize the lymph node yield and for better regional control.

Apical (central or D3) lymph nodal involvement is reported to be 0–11.1% in right-sided colon cancer [13, 14] and 0.3–8.6% have metastatic lymph nodes at the origin of the inferior mesenteric artery with left-sided colon and rectal cancer [15]. 0.8–2% may harbour skip metastasis from the epicolic node (pericolic or D1) to the main node (apical or D3) [16–20]. With adoption of CME techniques, Hohenberger [20] reports reduction in local recurrence rates from 6.5 to 3.6% and improvement in 5-year cancer-related survival rate from 82.1 to 89.1%. Thus, introduction of CME has improved oncologic outcomes.

Minimally invasive right colectomy should integrate and endorse the same oncological principles as that of open i.e. no-touch isolation technique, ligation of the vascular pedicles at their origin, for oncologic lymphadenectomy and adequate distal and radial margins [21].

Randomized trial JCOG-0404 [22] reported laparoscopic CME+CVL surgery was not non-inferior to open approach in terms of overall survival for patients with stage II or III colon cancer. Favourable outcomes of open CME have been replicated with a laparoscopic approach [23–25]. The safety and efficacy of laparoscopic CME approach have been repeatedly validated in the literature and the prevalence of laparoscopy in colon resection has reached a relative plateau. In the absence of the cost-effective conundrum associated with robotic surgery, the spectrum of indications for a robotic colon resection essentially mirror that of conventional laparoscopy. The aim of the current study is to discuss the essential components of appropriate CME, present technical details and to critically review the short-term oncologic outcomes of robotic CME with CVL for right-sided colon cancer.

## Materials and Methods

Fifty-two consecutive patients affected by right colon adenocarcinoma were operated between May 2016 to February 2020 with Da Vinci Xi platform (Intuitive Surgical Inc., Sunnyvale, CA, USA). All demographic, operative, pathological and postoperative recovery data were systematically collected by a prospectively maintained institutional colorectal specific database for statistical analysis. All operations were performed by a single surgeon with an extensive experience of about 30–40 annual open CRC resections over the past 25 years at Kidwai Memorial institute of Oncology, a regional cancer centre at Bengaluru, India.

### Eligibility Criteria

Eligible patients were those with histologically confirmed adenocarcinoma, mucinous adenocarcinoma, signet ring cell carcinoma, adeno-squamous carcinoma located in the caecum, ascending colon, proximal transverse colon and clinical T1–3, N0–2 and M0 lesions on contrast-enhanced computed tomogram of abdomen-pelvis-thorax.

cT4 or stage IV tumours, obstructing tumour, cancer associated with familial adenomatous polyposis or hereditary non-polyposis colorectal cancer, synchronous malignancies, lymphoma, presence of significant intra-abdominal adhesions limiting access to the colon and peritoneal deposits on staging/diagnostic laparoscopy were excluded.

### Adjuvant Chemotherapy

Adjuvant chemotherapy was administered for pT3 MSI stable tumours, pT4 and any T with positive lymph nodes. One of the three adjuvant chemotherapeutic protocols was administrated: (1) six cycles of 5-fluorouracil and leucovorin, (2) eight cycles of capecitabine and (3) 12 cycles FOLFOX from week 3–4 after surgery.

### Evaluation of Parameters

1. Conversion to open surgery was defined as the need for a laparotomy at any time to complete the entire surgical procedure after docking, excluding delivery of the specimen and extracorporeal anastomosis.
2. Clavien-Dindo (CD) classification system was used for analysing surgical complications.
3. Bowel obstruction/ileus was defined as the presence of at least three of the following six findings: nausea, vomiting, abdominal pain, abdominal distension, and absence of flatus and/or stool within the past 72 h, findings indicating obstruction on plain radiographic or contrast studies.
4. Anastomotic leak was defined as a disruption in the integrity of the anastomosis documented by a combination of clinical, endoscopic, radiologic and operative findings.

### Technique of Robotic Medial to Lateral Approach with CME + CVL for Right Colon Cancer

#### Step 1: Patient Positioning and Port Placement (Fig. 1)

**Fig. 1:**
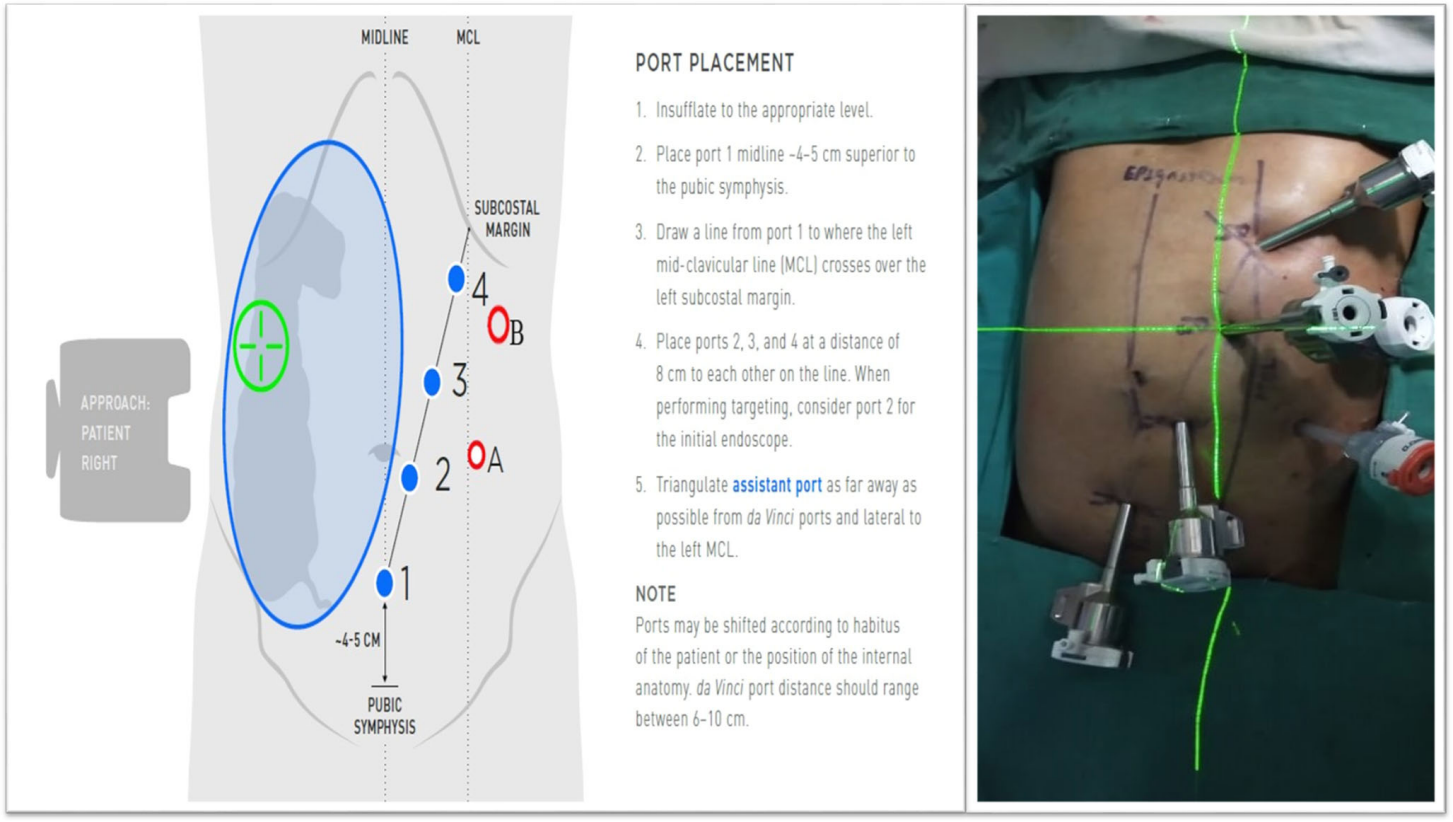
Port placement

The patient is placed in supine or a lithotomy position if intraoperative colonoscopy or transvaginal specimen extraction is planned. With the hands in abduction, the patient is secured to the operating table with a chest strap. After pneumo-peritoneum is created with a Veress needle in the palmer’s point, a 12-mm camera trocar in the left hypochondrium was used for performing a systematic staging laparoscopy to identify the extent of disease and to determine the feasibility of minimally invasive resection. Once the resectability was confirmed, additional ports are placed in a diagonal orientation extending 4 cm above the pubic symphysis and proceeding to the splenic flexure at 6- to 8-cm intervals as illustrated in Fig. 1. Diagonal orientation away from midline to the patient’s left provides in-line viewing and dissection of ileo-colic pedicle, middle colic vessel and access to a greater length of the transverse colon. Midline ports (placed along the linea alba) would lie directly above the ileocolic origin and might make its dissection more challenging. An additional 5-mm port can be triangulated between ports 2 and 3 depending on the patient’s body habitus. The patient is placed in 10–15° of Trendelenburg position with the 10–15° right side up. This allows for the small bowel and omentum to be displaced to the left upper quadrant, exposing the cecum and terminal ileum.

#### Step 2: Set up of Vision Cart and Robotic Cart (Fig. 2)

The vision stack is also located on the patient’s right side, by his right foot. The bedside assistant and the scrub nurse are situated to the patient’s left side The robot is then brought from the right side of the patient and docked onto the ports. The camera arm is attached to the port supero-lateral to the umbilicus (R-2) and adjusted to the point towards the hepatic flexure for targeting. The remaining robotic arms are secured to their respective ports. The Prograsp forceps is inserted into R-1, fenestrated bipolar into R-2 and mono-polar forceps into R-4 in the left upper quadrant. The platform is now set for the robotic operation to begin. Port hopping of the instruments can be performed to have two instruments to the right side of the camera when the need arises.

**Fig. 2:**
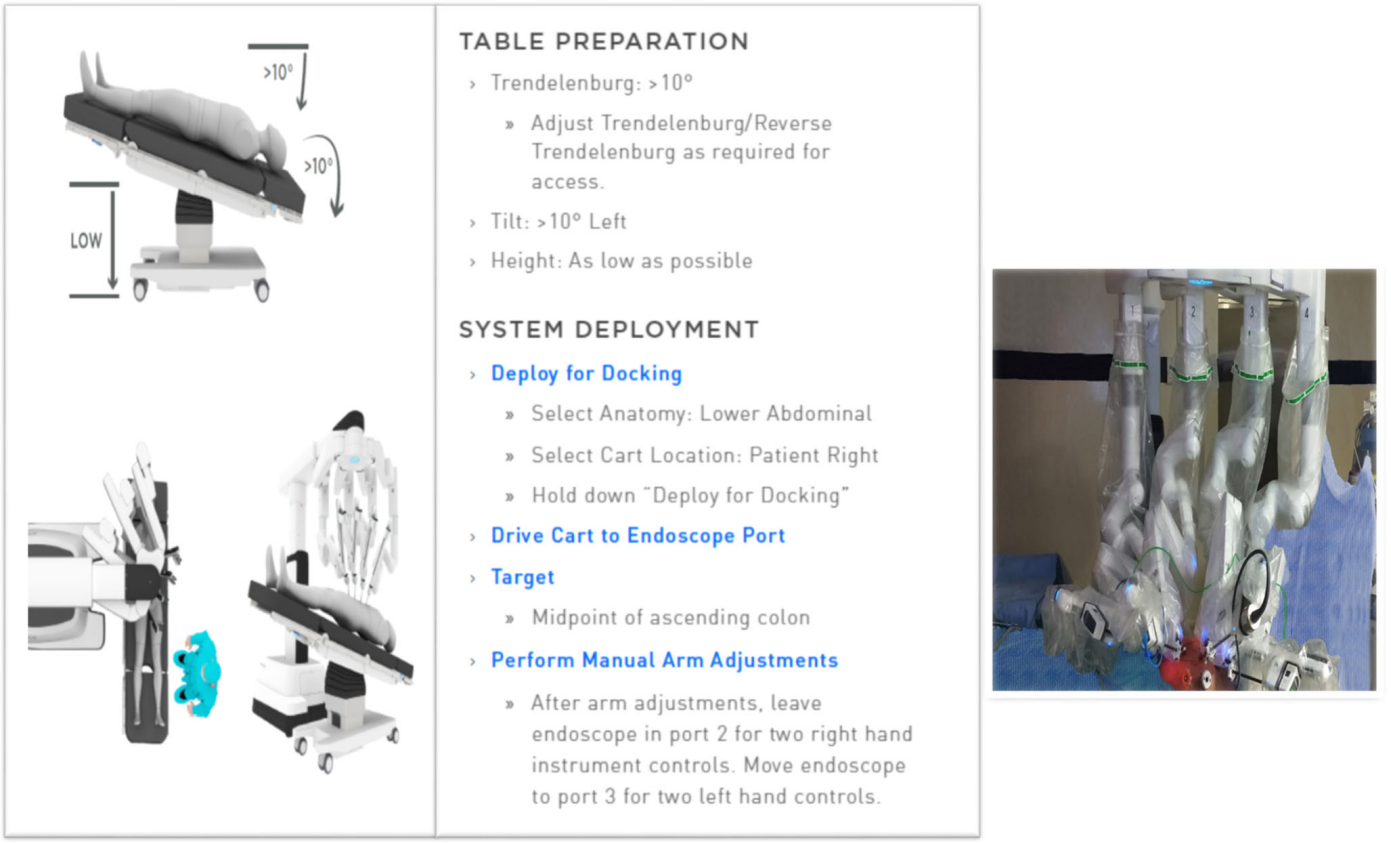
Docking

#### Step 3: Robotic Procedure

##### Medial-to-Lateral Dissection (Fig. 3)

Prograsp forceps (R-1) retracts the caecum/terminal ileum in supero-lateral direction to lift and delineate the ileocolic (IC) pedicle. The peritoneum layer of the mesentry below the IC pedicle is incised and an avascular retroperitoneal space dorsal to the vessels is created. Fenestrated bipolar (R-2) is passed under the IC pedicle and retracted towards the abdominal wall to facilitate development of the retroperitoneal space. The duodenum and pancreas are identified and displaced posteriorly. The dissection is further developed to identify right ureter, gonadal vessels and Toldt’s fascia and dissection is completed out to the lateral parietal attachments and to the underside of the hepatic flexure. The ascending colon to be left attached to the right paracolic gutter to keep it from falling medially.

**Fig. 3:**
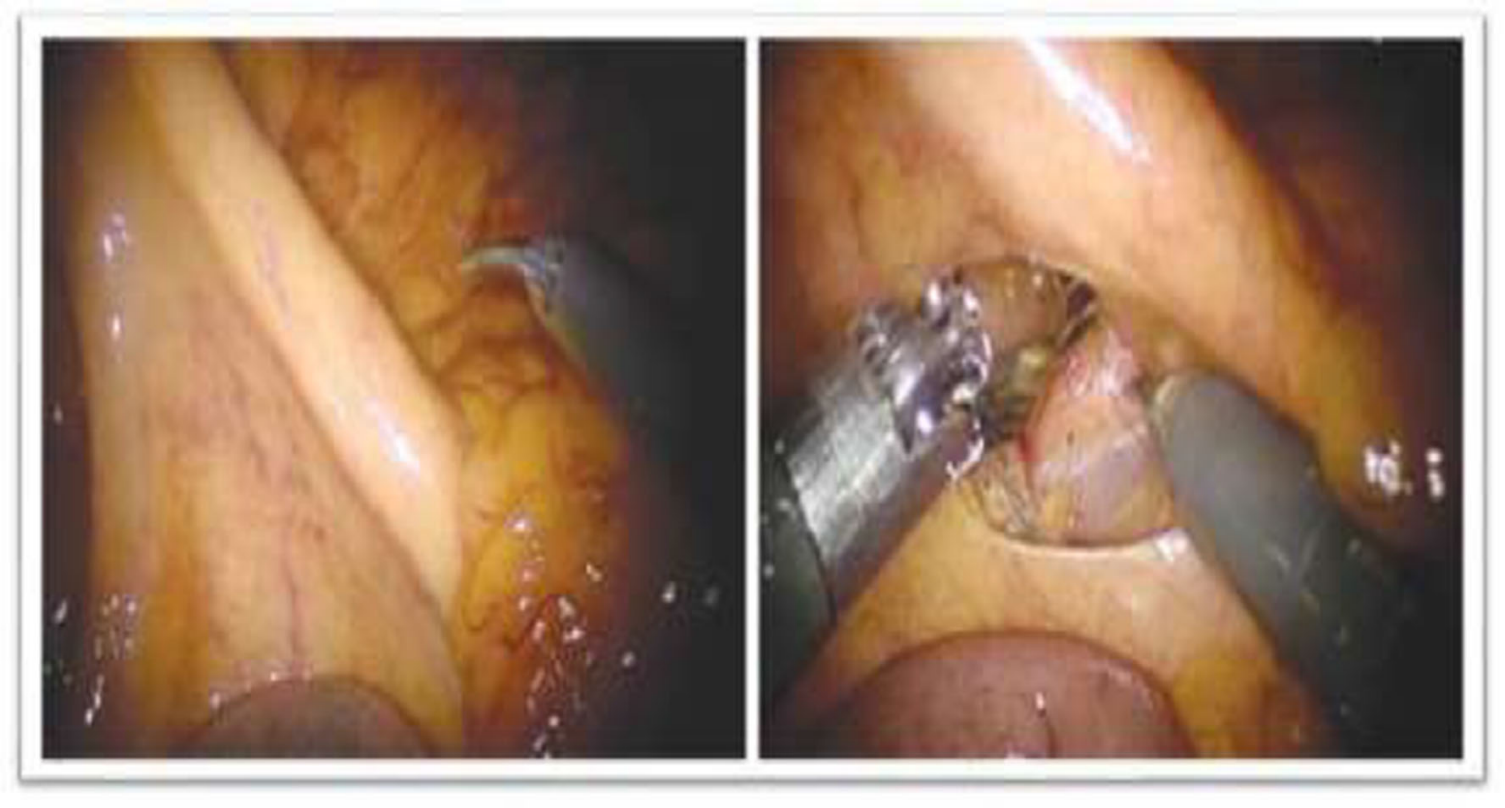
Medial to lateral dissection with ileocolic retraction and delineation of mesocolic plane from the retroperitoneum

### Central Vascular Dissection and Ligation

Port hopping is performed to facilitate inline viewing and dissection of the pedicles. The camera is hopped to the port in left iliac fossa (R-2) and the instruments are reconfigured, fenestrated bipolar in R-1, mono-polar in R-2 and Prograsp forceps in R-4. The IC pedicle is held by Prograsp (R-4) and dissection was performed along the vertical line of IC pedicle up to its origin until the superior mesenteric vein was visualized. The IC pedicle was divided at the root after securing between the hem-o-lock. The prograsp (R-4) retracts the transverse mesocolon cranially and dissection proceeds along the superior mesenteric vein axis to identify the right colic artery (if present), and colonic branch of the gastro-colic trunk. The colonic branch of the gastro-colic trunk is divided, preserving its gastric and pancreatic branches. The gastro-colic trunk has a number of anatomical variation and careful dissection is needed to avoid unintended vascular injury. Middle colic artery is exposed at its origin and the right branch of middle colic artery is divided after completing the lymphadenectomy. The root of the middle colic artery and vein were divided for the tumour located at the hepatic flexure and proximal transverse colon.

### Division of Gastro-colic Ligament and Lesser Sac Entry

The greater omentum of the transverse colon is retracted cranially by R-4 and transverse colon is retracted caudally by R-1 and the gastro-colic ligament is transected and the lesser sac was entered to join with the previous surgical plane of the medial dissection. The attachment of the hepatic flexure (hepato-colic ligatment) and the lateral peritoneum of the ascending colon are released.

### Specimen Extraction and Extracorporeal Anastomosis

Once complete colonic mobilization is performed, the robot is undocked and a small midline mini-laparotomy (6–7 cm) is made. Wound protector is applied and the mobilized right colon is exteriorized through this incision and resected with a liner stapler. A standard two-layer side to side iso-peristaltic ileocolic anastomosis is created either with PDS 4-0 or linear stapler. Indo-cyanine green with firefly mode, aided bowel transection or anastomosis, was not used in any of the patients.

### Data Collection and Statistical Analysis

All demographic, perioperative and post-operative recovery data were obtained from the prospectively maintained colorectal database. All statistical analyses were carried out with the Statistical Package for the Social Sciences version 21 (SPSS Chicago, IL, USA). Continuous variables were described as mean ± standard deviation unless stated otherwise.

### Ethics

The data of the present study were collected in the course of common clinical practice and, accordingly, the signed informed consent was obtained from each patient for any surgical and clinical procedure. The study protocol conforms to the ethical guidelines of the World Medical Association Declaration of Helsinki: Ethical Principles for Medical Research Involving Human Subjects adopted by the 18th WMA General Assembly, Helsinki, Finland, June 1964, as revised in Tokyo 2004. No approval of the institutional review committee was needed.

## Results

A total of 52 robotic-assisted right CME + CVL were performed by a single surgeon transitioning from open to robotic during the study period.

### Patient Characteristics

The baseline demographics of patients are summarized in Table 1. Median age was 55 years (range 22–70 years). Mean body mass index was 21.4 ± 3. Ascending colon involvement was noted in 75.15% (37) cases. Majority of the tumours were locally advanced and potentially resectable: cT3–82.69% (43) and node positive 63.46% (33) cases.

**Table 1:** Baseline characteristics of the patients

Variables	Numbers (%)
1. Median age	55 years (22–70 years)
Gender	
a. Male	31 (59.61%)
b. Female	21 (40.39%)
2. BMI	21.4 ± 3
ASA	
a. I–II	37 (71.15%)
b. III–IV	15 (28.85%)
Tumour location	
a. Ascending colon	37 (71.15%)
b. Hepatic flexure	08 (15.38%)
c. Proximal transverse colon	07 (13.46%)
Preoperative T stage	
a. T1–T2	09 (17.31%)
b. T3	43 (82.69%)
Preoperative N stage	
a. Node negative	11 (36.54%)
b. Node positive	33 (63.46%)

### Operative Outcomes

Table 2 illustrates operative parameters. 50/52 underwent complete robotic CME+CVL. 71.15% (37) cases underwent right hemicolectomy and 28.85% (15) cases required extended right hemicolectomy for hepatic flexure and proximal transverse colon tumours. Mean total operating time, docking and surgeon console time were 182 ± 66 min, 11 ± 6 min and 140 ± 22 min, respectively. Mean blood loss was 110 ± 90 ml. 02 (3.84%) cases required a conversion to open approach. Reasons for conversion are listed in Table 3.

**Table 2:** Operative outcomes

Variables	Numbers
1. Surgical procedure	52
a. Right hemicolectomy	37 (71.15%)
b. Extended right hemicolectomy	15 (28.85%)
2. Mean total duration of surgery	182 ± 36 min
3. Mean docking time	11 ± 6 min
4. Mean surgeon console time	140 ± 22 min
5. Mean blood loss	110 ± 90 ml
6. Conversion rates	02 (3.84%)

**Table 3:** Factors contributing for conversion to open approach

Unfavourable parameters	Numbers (%)
1. Hepatic flexure mass with peri-nephric fat infiltration	01 (1.92%)
2. Proximal transverse colon mass with duodenal infiltration	01 (1.92%

### Histopathologic Outcomes (Table 4)

Non-mucinous non-signet histology and signet ring histology were predominant and accounted for 40.38% (21) and 34.16%(18) cases, respectively. Majority of the tumours were grade II, 58% (29) cases. pT3 tumours were predominant and formed 78.84% (41) cases. Mean number of lymph-nodal retrieval was 31 ± 4.

**Table 4:** Histopathologic outcomes

Variables	Numbers (%)
1. Histopathology	*n* = 52
a. Adenoma	02 (3.84%)
b. Mucinous	11 (21.15%)
c. Non-mucinous	21 (40.38%)
d. Signet ring	18 (34.61%)
2. Grade	*n* = 50
a. I	5 (10%)
b. II	29 (58%)
c. III	18 (36%)
Pathological T stage	
a. pT1	00
b. pT2	07 (13.46%)
c. pT3	41 (78.84%)
d. pT4	02 (3.84)
Pathological nodal stage	
a. N0	11 (21.15%)
b. N1	19 (36.53%)
c. N2	22 (42.30%)
Tumour involved margins	
a. Positive margins	0 (0)
3. Mean number of retrieved lymph nodes	28 ± 4

### Postoperative Complications (Table 5)

Mean time to first flatus passage was 3.5 ± 1 day, time to resume to oral intake of liquids was 2 ± 0.5 day. Mean length of hospital stay was 7 ± 2 days. 1/52 (1.92%) case had an anastomotic leakage. 05/52 (9.61%) and 01/52 (1.92%) cases had CD grade IIIa and IIIb complications, respectively. There were no post-operative deaths.

**Table 5:** Postoperative outcomes and complications

Parameters	Numbers (%)
1. Mean time to first passage of flatus (days)	3.5 ± 1
2. Mean time to resume to oral intake of liquids (days)	2 ± 0.5
3. Mean length of hospital stay (days)	7 ± 2
4. Anastomotic leak rate	01 (1.92%)
5. Postoperative ileus	03 (5.76%)
6. Chyle leak	01 (1.92%)
7. Clavein-Dindo complications	
a. Grade I	39 (75%)
b. Grade II	07 (13.46%)
c. Grade IIIa	05 (9.61%)
d. Grade IIIb	01 (1.92%)
e. Grade IV	00 (0)
f. Grade V	00 (0)

## Discussion

According to the Japanese Society for Cancer of the Colon and Rectum, D2 lymphadenectomy refers to removal of epicolic, paracolic nodes (D1 Lymph nodes) along with intermediate nodes (D2 Lymph nodes) and D3 lymphadenectomy as removal of D3 nodes (Apical nodes) located at the origin of main pedicles in addition to D1 and D2 nodes [26]. Japanese D3 lymphadenectomy has been performed in many Asian countries which is based on similar principles to CME+CVL.

D3 dissection is generally recommended in patients with clinical stage II/III colon cancer [27]. D2 dissection as well as D3 are all acceptable in cT2N0 disease. However, we suggest that CME+CVL, which is similar to D3 dissection, may be useful even in cT2N0 disease because of limited accuracy of preoperative imaging, and D3 dissection can provide more accurate pathologic staging.

The Danish Colorectal Cancer Group demonstrated open CME surgery is oncologically superior to conventional non-CME surgery for patients with stage I–III colon cancer and reported a better 4-year disease-free survival with CME+CVL [28].

The laparoscopic approach offers the same quality of the resected specimen as the open CME+CVL for colon cancer. The laparoscopic approach has demonstrated superior perioperative results and non-inferior in long-term oncological outcomes [29]. Dissection of the lymph nodes around the superior mesenteric vessels, complex variable right colon vascular anatomy and technical limitations of laparoscopic instruments impose a technical challenge for laparoscopic CME+CVL, hence, the penetration of laparoscopic procedures is still considered slow.

The da Vinci surgical system has been developed to overcome such difficulties. The system is equipped with a three-dimensional camera, enables extra degrees of movement by using articulated instruments and is capable of physiological tremor filtration, hence, it can minimize the risk of injury to vessels and structures as well as provide oncological resection capability. Considering that performing CVL for right hemicolectomy along the axis of the superior mesenteric vein would involve a wide operative field from the right iliac fossa to the mid-transverse colon, the multi-quadrant capabilities of the Xi make it well suited for this task. However, there seems to be a relatively slow adoption of robotic approach in the CME technique for right-sided colon cancer.

CME+CVL has been advocated, but few series suggests a higher rate of intra-operative organ injuries (9.1% vs 3.6%, *P* < 0.001) and more severe non-surgical complications than with conventional resection, with an associated operative mortality rate of more than 6% in some published literature [30]. The present series critically reviews the feasibility and safety of the robotic CME and CVL, with short-term oncologic outcomes.

**Fig. 4:**
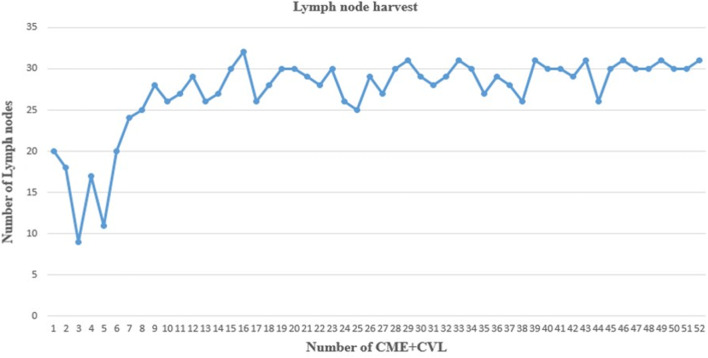
Lymph node yield with CME+CVL

From an oncological point of view, the nodal status is crucial. The number of harvested lymph nodes is a surrogate outcome of survival. The American Joint Committee on Cancer recommends the removal of at least 12 lymph nodes to ensure an accurate pathological staging of colon cancer after colectomy [31]. Suboptimal lymph nodal yield after radical colonic resection is reported to be in 33–78% [32–39]. In the present series, we found a mean number of harvested lymph nodes of 28 ± 4, with the progression of the surgeon’s experience an improvement in the number of lymph node harvest was noted. A significant improvement in the nodal harvest was observed after the initial seven cases (Fig. 4). The cases with less than 12 lymph nodes harvested were very limited (two cases). The improved lymph node yield with robotic approach is in accordance with other studies [40–42]. These observations suggest that robotic CME +CVL can be performed at the beginning of the robotic experience and is sound from an oncological point of view.

Wang et al. [43] reported that CME was associated with greater intraoperative blood loss and more postoperative morbidity than non-CME. In our study, CD grade IIIa and IIIb complications were reported only in 05 (9.61%) and 01 (1.92%) cases, respectively. Three cases had clinic-radiologically documented paralytic ileus which resolved with conservative management. One case had chyle leakage requiring total parenteral nutrition but recovered with conservative management. One case had an anastomotic leakage which was managed with an exploratory laparotomy and a diversion loop ileostomy. Regarding short-term outcomes, the data from present series demonstrate the technical feasibility and short-term safety of robotic CME in line with Bae et al. [44]. Estimated blood loss, length of hospital stay and the recovery of bowel was similar to Shixun et al.’s [45] systematic review. In the current study, the conversion rates to open approach is 3.84% (Table 3) which parallels the experience reported by Parisi et al. [46].

**Fig. 5:**
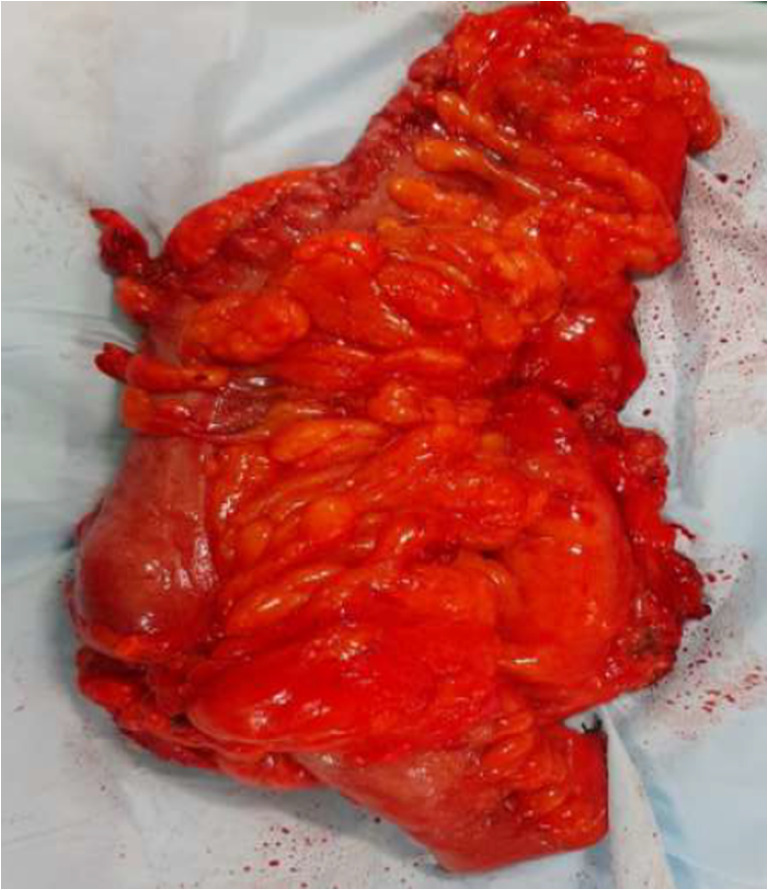
Robotic complete mesocolic excision specimen image showing intact meso-colon

Laparoscopic surgery cannot be performed alone by a single expert surgeon — the roles of the “camera assistant” to control vision and assistants to expose the surgical field in a limited space are critical. By assuming control of the endoscope, active instruments and arms, the robotic surgeon has the potential to control every aspect of the surgery. While the robot may not offer significant benefits to the experienced laparoscopic surgeon, the results of our study certainly support and enable a novice surgeon to master more complex procedures through without compromising the quality of surgery (Fig. 5) and safety of the patient.

Our study has few limitations such as it is a small and a retrospective series. However, the results are consistent with the previous and larger studies, a longer follow-up data is required to assess the long-term outcomes of local recurrence and cancer-free survival.

## Conclusion

The robot could help the transition of laparoscopically novice surgeons from open to minimally invasive colonic resections. Robotic CME+CVL may serve as an ideal procedure to begin the learning curve in robotic colorectal surgery, which can subsequently progress to rectal resection. In conclusion, although preliminary, this experience has shown that a robotic CME+CVL is not only safe and feasible but also associated with oncologically effective short-term outcomes.
